# Extraterritorial forays by great tits are associated with dawn song in unexpected ways

**DOI:** 10.1093/beheco/araa040

**Published:** 2020-05-20

**Authors:** Nina Bircher, Kees van Oers, Camilla A Hinde, Marc Naguib

**Affiliations:** 1 Behavioural Ecology Group, Wageningen University and Research, Wageningen, The Netherlands; 2 Department of Animal Ecology, Netherlands Institute of Ecology (NIOO-KNAW), Wageningen, The Netherlands; 3 Behavioural Ecology Research Group, Department of Biology, Anglia Ruskin University, Cambridgeshire, UK

**Keywords:** advertisement signals, dawn song, color ornaments, extrapair paternity, extraterritorial forays, radio tracking

## Abstract

Conspicuous male signals often play an important role in both attracting mates and deterring rivals. In territorial species with extrapair mating, female and male forays to other territories may be an important component underlying female choice and male mating success and might be influenced by male advertisement signals. Yet, whether off-territory foraying is associated with male signals is still not well understood. Here, we tested how female and male forays are associated with short-range visual and long-range acoustic signals (dawn song). We used an automated radio tracking system to follow the movements of wild great tits (*Parus major*) to other territories in relation to male dawn song, plumage ornaments, and extrapair paternity. We show that both sexes frequently forayed into others’ territories throughout the breeding period. Movements of both males and females were associated with male song but not with plumage ornaments. Contrary to our expectations, females stayed away from territories where males sang elaborately, whereas males were attracted to those territories. Moreover, neither female nor male forays were associated with the occurrence of extrapair offspring. Our results, thus, suggest that, although forays into other territories are associated with male dawn song, females may not be attracted and males not repelled by dawn song. This sheds a different light on the sex-specific effects of male advertisement signals, expanding the view on the selection pressures shaping such communication systems.

## Teaser

Conspicuous signals of many animals, like song in birds, may attract mates and repel rivals. We tracked the movements of wild great tits (*Parus major*) during an entire breeding season and show the opposite; females stayed away from territories of males with elaborate singing, whereas males were attracted. This suggests that the dawn song of male birds can have the opposite effect than commonly shown based on immediate responses, indicating a more complex role of song in communication systems.

## Introduction

The conspicuous signals of males in many animal species often serve a dual function in both intersexual selection and intrasexual competition (Berglund et al. 1996). These signals allow both females and males to gain important information on the motivation and quality of the signaler (Gil and Gahr 2002; Fischer et al. 2004; Hill and Farmer 2005; Behr et al. 2006), often using several different sensory modalities (Candolin 2003; Hebets and Papaj 2005). Acoustic signals, such as vocalizations, can be detected by receivers at greater distances than, for example, visual signals, like color ornamentation (Hebets and Papaj 2005). Using such long-range signals, receivers can assess a signaler from a distance without risking potentially costly physical interactions (Oliveira et al. 1998; Naguib and Wiley 2001; Peake et al. 2002). Thus, acoustic signals play a key role in facilitating or discouraging close-range associations between individuals and will affect whether or not information from short-range signals will be accessible (Snijders and Naguib 2017). In territorial species like, for example, several well-studied songbirds, the conspicuous advertisement signals of males have been shown to repel other males (Krebs et al. 1978; Nowicki et al. 1998; Snijders et al. 2017a) but attract females (Chiver et al. 2008; Snijders et al. 2017a). Specifically, the high singing activity at dawn (“dawn song”) that males engage in throughout the breeding season may play an important role in keeping away territory intruders and attracting (extrapair) mates (Staicer et al. 1996; Gil and Llusia 2020). However, our understanding of the behavioral responses of males and females in relation to long-range signaling stems mostly from playback studies focusing on immediate spatial responses or from correlational studies linking male song traits to reproductive success (Catchpole and Slater 2008). Especially, in species with extrapair mating, where either the female or male has to foray off-territory (“extraterritorial foray”) in order to search for potential extrapair mates (Kempenaers et al. 1992; Schlicht et al. 2015), the behavioral response to male singing is still not well understood (Bircher and Naguib 2020).

Both females and males have been shown to engage in extraterritorial foraying (Neudorf et al. 1997; Kleven et al. 2006; Akçay et al. 2012; Celis-Murillo et al. 2017). Such forays could have multiple, nonexclusive functions: individuals may foray into other territories to assess, for example, territory quality and breeding success to optimize their own future reproductive and settlement decisions (Doligez et al. 2004; Ward 2005) or in search for food and nesting material (Westneat 1993). There is evidence in some species that females foray more often when they are fertile (Neudorf et al. 1997; Double and Cockburn 2000; Chiver et al. 2008), indicating that these forays might be used to copulate with extrapair mates. Evidence from radio tracking (Kleven et al. 2006) and from monitored visits to other nest-boxes recorded with pit tags (Schlicht et al. 2015) shows that male foraying effort predicts the occurrence of extrapair offspring and is correlated with success in obtaining extrapair fertilizations. Males in some species also forayed more often into other territories when females are fertile (Pedersen et al. 2006; Akçay et al. 2012), suggesting that they actively seek out potential extrapair mates when foraying. However, foraying could also be costly for both males and females as it may lead to aggressive encounters with territory owners (Dale and Slagsvold 1995). Moreover, females may risk losing parental care by their social mate when engaging in extrapair copulations, whereas males may face a trade-off between foraying in pursuit of extrapair copulations and mate guarding to avoid being cuckolded (Westneat and Stewart 2003). Thus, in territorial species with extrapair mating, females could use long-range signals, such as dawn song, to decide from a distance where to foray. In some species, female forays take place specifically during a time of day with high male singing activity, which would allow females to sample male song particularly well (Double and Cockburn 2000; Roth et al. 2009). Female black-capped chickadees were observed to sometimes copulate with extrapair mates during twilight at dawn (Mennill et al. 2004) and, in great tits, females with extrapair young emerged earlier from their nest-boxes at dawn compared with females without extrapair offspring just before egg laying (Halfwerk et al. 2011), suggesting that the time at dawn might be important for extrapair behavior. Similarly, males might use acoustic or visual signals to assess the fighting ability or dominance of other males and decide where there is a better chance to cuckold the territory owner. To date, most studies on foraying behavior have focused on either males or females, were restricted due to time consuming behavioral observations and manual radio tracking, or did not include both acoustic and visual traits that may play a role in prospecting behavior.

Here, we took a comprehensive approach to determine the relation between male and female extraterritorial forays and male signals, integrating both acoustic and visual signaling and genetic analyses using wild great tits (*Parus major*) as a model species. We quantified male vocal (dawn singing) and plumage (yellowness and size of the black breast stripe) ornaments. We used an automated tracking system to track male and female spatial behavior continuously throughout the breeding season to determine how foraying associates with male signaling and whether or not it is connected to the occurrence of extrapair offspring. We determined throughout the whole breeding season 1) the frequency and number of males and females intruding into another territory and 2) the time males and females spent on another territory in relation to the resident male’s song and plumage traits. We expected that females foray mostly when they are fertile and are attracted to territories of males with more elaborate song traits. Conversely, we predicted males to stay away from territories of other males with more elaborate signals and to foray less often during the time their mate is fertile to avoid getting cuckolded. We expected acoustic, long-range signals (dawn song) to be more important than visual and short-range signals in attracting or repelling other individuals from territories. Finally, we predicted that broods of females that are foraying frequently, broods of males that are foraying frequently when their mate is fertile (and therefore would mate guard less), and broods in territories that are often intruded by other males are more likely to contain extrapair offspring.

## Materials and methods

### Study site and general field methods

This study was conducted in 2016 in Westerheide, a mixed deciduous forest near Arnhem, The Netherlands, with approximately 100 breeding pairs of great tits breeding in nest-boxes per year. From late March to late June, we checked nest-boxes about once per week to determine the start of egg laying, start of incubation, hatching date, and fledging success. We caught parents during chick rearing using spring traps when chicks were 10 days old (hatching = day 0) and measured weight, length of tarsus, and wing length (length of the third primary; P3) to the nearest mm. We collected two blood samples of approximately 10 µL from the brachial vein from each parent. When chicks were 14 days old, we ringed them with a unique aluminum ring and measured tarsus length and weight. We collected one blood sample of approximately 10 µL from the brachial vein from each chick.

### Radio tracking and quantifying forays

We used an automated radio tracking system called “Encounternet” (Encounternet LLC, Portland, OR) to continuously track movements of males and females during the breeding season (Mennill et al. 2012; Snijders et al. 2014, 2017a). Encounternet consists of roaming nodes (tags) transmitting an individual ID code every 5 s and base nodes (receivers) that were distributed throughout the study site. Receivers store the ID code, time stamp, and signal strength (Received Signal Strength Indication-RSSI) value for every signal within up to 90 m from the transmitter. On March 22, 2016 we tagged 79 birds (41 females and 38 males). Birds were caught while roosting in their nest-boxes and equipped with a radio tag of approximately 1.2 g using a leg loop harness, then released back to their nest-box (Snijders et al. 2017b). We received permission for all bird handling procedures in this study by the Dutch legal entity Dier Experimenten Comissie.

We placed Encounternet receivers up to 5 m from nest-boxes at a height of approximately 2 m to monitor tagged birds entering the area around a nest-box. In the area we monitored, 58 nest-boxes were used by great tit pairs for breeding but, due to technical difficulties with some of the receivers, data were collected for only 38 territories. We used signal strength values (RSSI) stored by receivers to estimate the distance between detected tags and receivers (Mennill et al. 2012) and, subsequently, excluded all logs that were determined to be further away from a nest-box than 15 m. We estimated the cutoff signal strength value at 15 m using a RSSI-distance regression based on measures from nine calibration transects conducted at different areas within the study site. To do so, we positioned tags at six different distances (2, 10, 20, 30, 40, and 50 m), three different heights (ground level and 2 and 6 m) with different antenna angles and either moved them slightly or held them still to simulate birds in different positions. We calibrated all receivers before deployment to account for between-receiver variation in detection sensitivity. For details on the calibration of the radio tracking system, see the Supplementary Material. We choose a radius of 15 m, so we could assign a received signal to a nest-box (the smallest distance between neighboring boxes in our study area was 30 m). Using a sliding window approach, we treated a bird as present near a nest-box as long as its tag was logged by the respective receiver at least three times during any 30-s time window (i.e., the receiver logged 50% of the signals sent out by the tag in 30 s). This way we excluded single logs resulting from birds just passing through the territory. Following this rule, we considered any time a bird was present near a nest-box that was not its own breeding box as an extraterritorial foray. We were, thus, able to detect whether, when, and for how long tagged individuals were present in the vicinity of another pair’s breeding area. However, our data does not provide information about which other individuals they have close encounters with (e.g., whether foraying intruders ever approach the opposite sex member of the resident pair).

Of the 79 birds tagged in March, 59 (31 females and 28 males) also bred in our study site, 49 of them in the area covered by receivers. We removed tags when the tagged birds’ chicks were 14 days of age. Tagged birds were tracked for an average of 31 ± 20 days. We used data obtained from March until the end of May, thus covering the majority of the breeding period, only excluding the day after tagging. For analysis, we divided the tracking period into five stages relative to the onset of egg laying (day 0): prefertile (from the beginning of tracking until day −8, fertile (the week before the first egg from day −7 until day −1; Birkhead 1992), egg laying (day 0 until the last egg), incubation (day after last egg until hatching) and nestling stage. During the nestling stage we monitored only the first 5 ± 4 days. Due to technical difficulties with some receivers, the other stages (prefertile, fertile, egg laying, and incubation) were fully monitored only for 23 nest-boxes but, for all 38 boxes at minimum, the egg laying stage was fully covered. We divided the day into early morning (5:00 AM to 7:00 AM, which includes the twilight period before sunrise when males are singing), morning (7:00 AM to 12:00 noon), afternoon (12:00 noon to 6:00 PM), and evening (6:00 PM to 8:00 PM). We excluded visits logged during the later evening and night (8:00 PM to 5:00 AM) as they seemed to be mostly caused by birds sleeping close to other boxes during the prefertile period.

### Acoustic recordings and measurements

We recorded male dawn song on several days during the fertile period of their mate using time-programmed Song Meter SM3 recorders (Wildlife Acoustics, Inc. Maynard, MA) mounted above the nest-boxes. We selected the best-quality recording for each male (highest signal-to-noise ratio) for analysis. All recordings selected for analysis were recorded just before egg laying or during early egg laying (day −3 to day 4 with day 0 being the day of the first egg laid). Recordings were analyzed in Avisoft-SASLab Pro version 5.2.10 (Avisoft, Berlin, Germany). We measured the start and end time of the dawn song in minutes before sunrise, the average song duration (seconds), song rate (songs per minute), dawn song duration (minutes), proportion of time spent singing, and the repertoire size for the entire dawn song. All six measures have previously been suggested to indicate male quality or be associated with female choice in great tits or other species (Hasselquist et al. 1996; Gil and Gahr 2002; Poesel et al. 2006). We defined the start of the dawn song as the time a bird sang its first song and the end as the time when a bird stopped singing for longer than 7 min (Naguib et al. 2019). To determine repertoire size, we followed existing song type categorization criteria for great tits (McGregor et al. 1981). All measures were taken by the same person, who was blind to other information about the individual (tracking data or paternity results) at that point. Because recordings were made with automated, stationary recorders, our analysis is restricted to the part of the dawn song a male sang in the vicinity of the nest-box. We recognize that by only measuring song characteristics from one day, we do not take within-individual variation in singing behavior during the season into account. However, other studies have shown that single dawn song recordings can provide important information about the singer (see, e.g., Otter et al. 1997; Poesel et al. 2006). Moreover, the start of dawn song and song rate were repeatable between the early and late egg laying stages in the same study population (Snijders et al. 2015), and start of dawn song and repertoire size did not differ between some breeding stages (before and during egg laying; Naguib et al. 2019).

Males started their dawn song on average 30.6 ± 1.4 min (mean ± standard error [SE]) before sunrise and sang for 28.2 ± 2.4 min (mean ± SE) with a mean song rate of 6.4 ± 0.4 songs (mean ± SE) per minute, song duration of 2.3 ± 0.09 s (mean ± SE), repertoire size of 3.1 ± 0.3 song types (mean ± SE), and proportion of time spent singing 32.2 ± 1 % (mean ± SE). We performed a principal component analysis (PCA) using IBM SPSS Statistics v. 23.0 (IBM Corp., 2013, Armonk, NY) with Varimax rotation and Kaiser Normalization, including all three principal components with eigenvalues >1. For further analysis, we used the first three components, which accounted for 82% of the total variation in the six song measures. PC1 was positively associated with dawn song duration, start time of the dawn song, and repertoire size. Males with a high PC1 score, therefore, sang for longer at dawn, started their dawn song earlier (as we measured start time in minutes before sunrise so that higher values indicate an earlier start), and sang a larger number of different song types during their dawn song. PC2 was positively associated with the proportion of time spent singing and song rate; thus, males with a high PC2 score sang more during their dawn song. PC3 was positively associated with mean song duration; thus, males with high PC3 scores sang, on average, longer songs during the dawn chorus (see Table 1 for all factor loadings on PC1, PC2, and PC3).

**Table 1:** PCA loadings of song measures. PC1 was positively associated with dawn song duration (factor loading = 0.88), start time of the dawn song (factor loading = 0.81), and repertoire size (factor loading = 0.78). PC2 was positively associated with the proportion of time spent singing (factor loading = 0.90) and song rate (factor loading = 0.81). Mean song duration was positively associated with PC3 (factor loading 0.98)

	PC1	PC2	PC3
Dawn song duration	**0.88**	−0.42	0.10
Start time of dawn song	**0.81**	−0.24	0.17
Repertoire size	**0.78**	−0.28	−0.11
Proportion of time spent singing	0.12	**0.90**	0.37
Song rate	−0.27	**0.81**	−0.46
Mean song duration	0.03	0.06	**0.98**

### Plumage measurements

When adults were caught during the breeding season, we took a photo of the breast stripe and collected samples of the yellow breast feathers to quantify breast stripe size and yellowness of the plumage for males. Both plumage traits have been suggested to be important indicators of male quality in the great tit (Norris 1993; Senar et al. 2008). We took photos with a Coolpix L31 camera (Nikon Corporation, Tokyo, Japan) mounted on a camera stand in standardized distance and angle, holding the bird with the crown and legs touching the background on which was a 1-cm^2^ grid sheet (Figuerola and Senar 2000). We measured the size of male breast stripes in square centimeters from where the ventral stripe widens into a throat patch to the posterior end of the stripe with the image analysis software *ImageJ* v. 1.45s (Abramoff et al. 2004).

We collected feather samples from the yellow patches from each side of the breast stripe and measured plumage reflectance following the methods in (Quesada and Senar 2006).The samples of yellow breast feathers consisted of at least 12 feathers (6 from each side), a number that has been shown to produce the same color measurements as when measuring directly on the bird (Quesada and Senar 2006). We stacked the feathers on a black velvet surface, superimposing four layers of three feathers to imitate the plumage surface of a bird and obtained reflectance spectra using a spectrometer (JAZ, Ocean Optics, Dunedin, FL) with a xenon light source (JAZ-PX, Ocean Optics) and a bifurcated fiber-optic probe. The probe was fitted with a cylindrical probe holder to exclude ambient light and standardize the measuring distance to 0.8 mm and we held it perpendicular to the feather sample for measuring. We obtained reflectance measures with the program SPECTRASUITE v. 2.0.162 (Ocean Optics) in reference to a white tile surface. In total, we took nine readings per sample, reshuffling the feathers after every third reading. Each reading itself was the average of 12 scans of 40 ms duration. Using the program TETRACOLORSPACE v. 1b BETA (Stoddard and Prum 2008), we then calculated the average photon catch of each color cone type; ultraviolet sensitive or violet (UVS), short-wavelength sensitive or blue (SWS), medium-wavelength sensitive or green (MWS), and long-wavelength sensitive or red (LWS) for the average avian visible spectrum (Stoddard and Prum 2008). TETRACOLORSPACE uses data on cone sensitivity to model the signal from a bird’s perspective. We then used these average cone catches of each sample to calculate its SWS ratio, a measure of intensity of the yellow plumage (Evans et al. 2010) using the formula 3^−1^(UVS + MWS + LWS)/SWS.

### Extrapair paternity

All collected blood samples were suspended in Eppendorf tubes containing 1 mL of Queen’s lysis or cell lysis buffer. We extracted and amplified DNA with the FavorPrep 96-well Genomic DNA Kit (Favorgen Biotech Corporation, Ping-Tung, Taiwan) and the QIAGEN multiplex PCR kit (QIAGEN GmbH, Hilden, Germany) following the manufacturer’s protocol. To determine paternity of chicks, we used five microsatellite markers: *PmaTAGAn71*, *PmaGAn27*, *PmaTGAn33*, *PmaC25*, and *PmaD105* (Saladin et al. 2003) in one multiplex PCR. PCR products were run on an ABI 3130 genetic analyzer (Applied Biosystems, Foster City, CA) with a molecular size standard (GeneScan 500-LIZ, Applied Biosystems). We used GeneMapper v. 4.0 (Applied Biosystems) to determine the sizes of the PCR products and derive the genotype for each individual. We determined whether a chick was within pair or extrapair with CERVUS v. 3.0.7 (Marshall et al. 1998) testing all chicks against their putative fathers using the following simulation parameters: 98% of loci typed, error rate 0.01%, 10 000 cycles, and two candidate parents. We treated chicks as extrapair if there were two or more mismatches with the putative father and the putative father was not the social father according to the analysis in CERVUS (significant trio LOD i.e., logarithm of the odds score). For two nest-boxes, we did not catch the male during the field season and inferred the putative father genotype based on the genotypes of the chicks and the mother and, then, used the inferred genotype to identify the father among the tagged males. We confirmed the identity of these two males using the tracking data at the respective nest-boxes. Two nests, in which chicks did not hatch or died before sampling, were not included in the analysis. We determined the extrapair status of 441 out of 443 sampled chicks of 73 broods. For two chicks (in separate nests), we did not have a blood sample. The combined exclusion probability for all microsatellites was >99.9%. Two of our loci significantly deviated from the Hardy–Weinberg equilibrium when the genotypes of all individuals in the analysis were included (*PmaD105*: χ ^2^ = 24.81, degrees of freedom [df] = 10, *P* = 0.006; *PmaTAGAn71*: χ ^2^ = 29.58, df = 10, *P* = 0.001). This was likely due to the family structure of the data.

### Statistical analyses

All statistical analyses were done in R v. 3.4.3 (R Core Team 2019). We analyzed all data with generalized linear models and generalized linear mixed models (GLMMs) using the R packages *lme4* (Bates et al. 2015) and *glmmTMB* (Brooks et al. 2017) and checked model assumptions using diagnostic plots created with the R package *DHARMa* (Hartig 2019). We determined the significance of fixed effects with likelihood-ratio tests and used stepwise backwards elimination, starting with the least significant variable, to obtain minimal adequate models. Fixed effects used as control factors (lay date relative to the population median and average distance to other boxes included in the tracking study), as well as random factors, always remained in the minimal adequate model independent of significance.

### Resident male traits and visiting behavior of males and females

We quantified visits by females and males as number of forays to a nest-box area per hour and number of unique individuals foraying to a nest-box area per hour, treating each unique hour as a separate data point. For all hours for which we had no individuals visiting a nest-box, we added a 0 for both number of visits per hour and number of individuals visiting per hour. We then tested whether there is a correlation between the number of visits, the number of visiting individuals, the duration of visits to a nest-box (dependent variables), and the traits of the resident male (fixed variables: PC1, PC2, yellowness, and breast stripe size). Measures of all four male traits were available for 24 of the 38 boxes with tracking data and we used this subset of our data for all models. We included the average distance to other nest-boxes and lay date relative to the population median as fixed factors to account for possible differences in visiting behavior to certain boxes because of their central or edge locations and early or late breeding start. We added nest-box ID as random factor to correct for multiple observations per nest-box and ran a separate model for female and male visits. We first fitted Poisson GLMMs for both the number of visits and the number of visitors per hour. We tested these Poisson GLMMs for overdispersion and zero inflation using the respective tests provided by the R package *DHARMa* (Hartig 2019). Because we found evidence for zero inflation in the counts of visits to a nest-box per hour, we then fitted a zero-inflated Poisson GLMM to that data using the R package *glmmTMB* (Brooks et al. 2017). We used a Poisson GLMM to analyze the number of individuals visiting a nest-box per hour and log-transformed visit duration values to use a linear mixed model for the visit duration data.

### Timing of forays and traits of foraying individuals

We quantified the number of forays made by each individual per hour. All hours during which the individual was tracked but no foray was detected were scored as 0. We then tested whether the number of forays per hour and the duration of forays made by an individual correlated with the time of the day or the breeding stage (fixed variables). For males, we used the breeding stage of their mate. We knew the breeding stage for every foray for 24 males and 26 females and used the respective data subset for all models. We added lay date relative to the population median and average distance to other nest-boxes as fixed variables to control for potential spatial or temporal patterns and individual ID as random factor to account for several observations per individual. We first fitted a Poisson GLMM to the number of forays per hour but found evidence for zero inflation using the respective test provided by the R package *DHARMa* (Hartig 2019). We thus used a zero-inflated Poisson GLMM to model the number of forays per hour using the R package *glmmTMB* (Brooks et al. 2017). We log-transformed visit duration values to use a linear mixed model for the duration data. We tested males and females using separate models. Additionally, we quantified the number of different nest-box areas visited by foraying females and males per day: we added a 0 to the data set for each day an individual was tracked but not detected in any nest-box area. We then tested whether the number of areas visited was associated with breeding stage (fixed variable), adding lay date relative to the population median and average distance to other nestboxes as control variables and individual ID as random factor in a Poisson GLMM, testing males and females separately. Because our results showed that males make the longest forays early in the morning, we subsequently also tested for a relationship between dawn song characteristics (PC1 and PC2) and duration of male forays early in the morning. We used a linear mixed model with either PC1, PC2 or PC3 as fixed factor (in three separate models) and the log-transformed foray duration values as dependent variable. In each model, we controlled for lay date relative to the population median and average distance to other nest-boxes and included male ID as random factor.

### Extrapair paternity and forays

We quantified the average number of forays made per hour by the female and male during the fertile and egg laying stages, the average number of visits by other males received per hour, and the occurrence of extrapair offspring (0/1) for each nest-box. Due to the small number of nest-boxes with a receiver that had both parents tagged, we used separate binomial linear models to test whether either the forays by the female, the forays by the male, or visits by other males predict the occurrence of extrapair offspring. We included the lay date relative to the population median for each box and the average distance to other boxes as control factors in each model.

## Results

We collected tracking data of 66 birds (34 females and 32 males), accumulating a total of 30 325 forays (13 995 by females and 16 330 by males) to 38 monitored nest-box areas. Visits had an average duration of 66 s and the longest visit lasted 1.9 h. Females forayed on average 0.45 ± 0.004 (mean ± SE) times per hour and 6.73 ± 0.07 (mean ± SE) times per day. Males made on average 0.59 ± 0.005 (mean ± SE) forays per hour and 10.17 ± 0.09 (mean ± SE) forays per day.

## Receiving forays

### Males with a higher PC1 score received fewer visits by females

**Figure 1:**
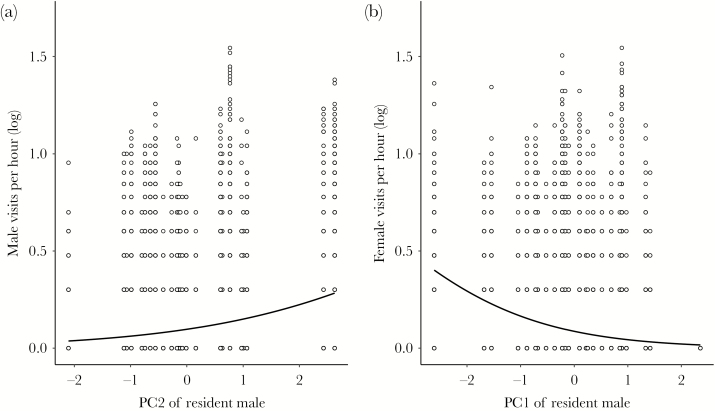
Associations between song traits (PC1 and PC2) of the resident male and the number of male and female visits to the respective nest-box area. Males with a higher PC2 score received more visits by other males. Males with a higher PC1 score received fewer visits by females. We added 1 to counts of visits per hour in order to present them on a log scale. Data based on 20 265 observation hours and 24 resident males, observations with exactly the same values are plotted as one point.

Resident males with higher PC1 scores (earlier start, longer dawn song, and larger repertoire) received fewer female visits per hour and visits by fewer females per hour (number of visits per hour: χ ^2^ = 8.12, *P* = 0.004; see Figure 1; number of visitors per hour: χ ^2^ = 7.72, *P* = 0.006, *N* = 20 265 observation hours, 24 resident males). The resident male’s breast stripe size, yellowness, PC2, and PC3 score were not associated with the number of female visits per hour and the number of female visitors per hour (visits per hour: breast stripe size: χ ^2^ = 0.25, *P* = 0.62, yellowness: χ ^2^ = 0.06, *P* = 0.80, PC2: χ ^2^ = 0.94, *P* = 0.33, PC3: χ ^2^ = 1.69, *P* = 0.19; visitors per hour: breast stripe size: χ ^2^ = 0.15, *P* = 0.70, yellowness: χ ^2^ = 0.05, *P* = 0.82, PC2: χ ^2^ = 0.72, *P* = 0.40, PC3: χ ^2^ = 2.48, *P* = 0.12). Males with a high PC3 score (longer song duration) tended to receive shorter visits by females (χ ^2^ = 3.29, *P* = 0.07), but the duration of female visits was not associated with any of the other measured male traits (breast stripe size: χ ^2^ = 0.07, *P* = 0.8, yellowness: χ ^2^ = 0.15, *P* = 0.7, PC1: χ ^2^ = 0.54, *P* = 0.46, PC2: χ ^2^ = 0.06, *P* = 0.8, PC3: χ ^2^ = 2.58, *P* = 0.11; see Table 2 and Supplementary Tables S2 and S4).

**Table 2:** Traits of resident male and number of female visits per hour. Table lists all factors included in a zero-inflated Poisson GLMM with log link function. The dependent variable was the number of visits per hour to a resident male’s nest-box area (*N* = 20 265 observation hours, 24 resident males) by females. Nest-box area was included as random factor (var ± standar deviation: 1.42 ± 1.19). Using backward elimination, the estimate and SE of the last model in which a factor was included are given. The test statistic (χ ^2^), df, and significance (*P* value) given were determined using likelihood-ratio tests. The factors distance (average distance of a resident male’s nest-box to other nest-boxes) and relative lay date of the resident male’s brood were included in the last model independent of significance

	Estimate	SE	χ ^2^	df	P value
Intercept	−0.12	0.25	—	—	—
PC1	−0.74	0.25	8.12	1	**0.004**
Distance (scaled)	−0.09	0.22	—	—	—
Relative lay date (scaled)	−0.11	0.26	—	—	—
Dropped terms					
Yellowness	−0.46	1.81	0.06	1	0.80
Stripe	−0.09	0.18	0.25	1	0.62
PC2	0.21	0.22	0.94	1	0.33
PC3	0.36	0.27	1.69	1	0.19

### Males with a higher PC2 score received more and longer visits by other males

Resident males with a high PC2 score (higher song rate and proportion of time spent singing) received more and longer visits by more males (visits per hour: χ ^2^ = 5.86, *P* = 0.02; visitors per hour: χ ^2^ = 5.03, *P* = 0.03; *N* = 20 265 observation hours, 24 resident males; visit duration: χ ^2^ = 12.68, *P* < 0.001; *N* = 10 253 visits, 24 resident males). Resident males with higher PC3 scores (longer song duration) received—and residents with higher PC1 score (earlier start, longer dawn song, and larger repertoire) tended to receive—shorter visits by other males (PC3: χ ^2^ = 7.19, *P* = 0.007; PC1: χ ^2^ = 3.24, *P* = 0.07), but neither PC3 nor PC1 were associated with the number of visits per hour by other males or the number of males visiting per hour (visits per hour: PC3: χ ^2^ = 1.48, *P* = 0.22; PC1: χ ^2^ = 0.08, *P* = 0.78; visitors per hour: PC3: χ ^2^ = 1.33, *P* = 0.25; PC1: χ ^2^ = 0.17, *P* = 0.68).

Neither breast stripe size nor yellowness of resident males was correlated with the number of male visits per hour, number of male visitors per hour, or the duration of visits by other males (number of visits: breast stripe size: χ ^2^ = 0.18, *P* = 0.67, yellowness: χ ^2^ = 0.04, *P* = 0.85; number of visitors: breast stripe size: χ ^2^ = 0.12, *P* = 0.68, yellowness: χ ^2^ = 0.02, *P* = 0.88; duration: breast stripe size: χ ^2^ = 0.01, *P* = 0.92, yellowness: χ ^2^ = 0.57, *P* = 0.45; see Table 3 and Supplementary Tables S1 and S3).

**Table 3:** Traits of resident male and number of male visits per hour. Table lists all factors included in a zero-inflated Poisson GLMM with log link function. The dependent variable was the number of visits per hour to a resident male’s nest-box area (*N* = 20 265 observation hours, 24 resident males) by males. Nest-box area was included as random factor (var ± standard deviation: 0.99 ± 0.99). Using backward elimination, the estimate and SE of the last model in which a factor was included are given. The test statistic (χ ^2^), df, and significance (*P* value) given were determined using likelihood-ratio tests. The factors distance (average distance of a resident male’s nest-box to other nest-boxes) and relative lay date of the resident male’s brood were included in the last model independent of significance

	Estimate	SE	χ ^2^	df	*P* value
Intercept	−0.13	0.21	—	—	—
PC2	0.50	0.19	5.86	1	**0.02**
Distance (scaled)	−0.01	0.18	—	—	—
Relative lay date (scaled)	−0.13	0.21	—	—	—
Dropped terms					
Yellowness	−0.30	1.52	0.04	1	0.85
PC1	−0.06	0.19	0.08	1	0.78
Stripe	−0.07	0.16	0.18	1	0.67
PC3	−0.28	0.23	1.48	1	0.22

## Making forays

### Foraying activity of males and females is associated with breeding stage and time of day

**Figure 2:**
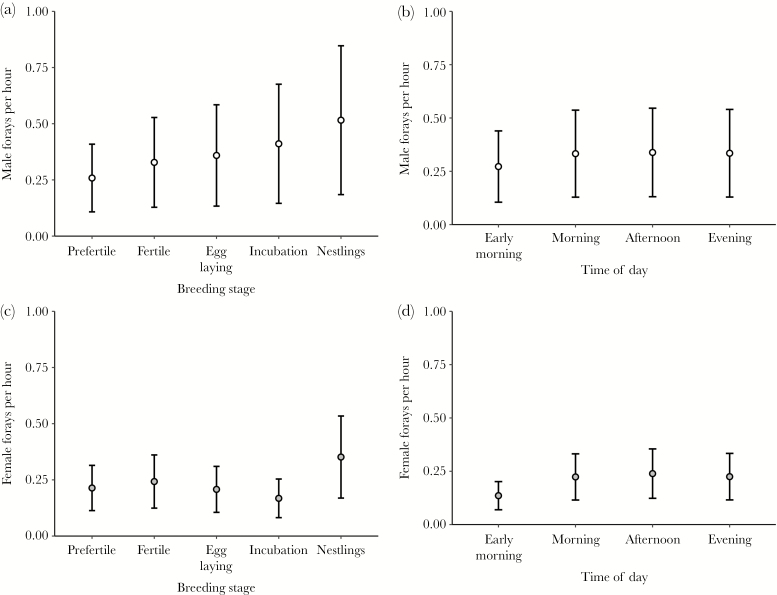
Foraying activity (number of forays made per hour) of males and females over the breeding season and day. Both males (white) and females (grey) forayed least often during the early morning (b and d) and most often during the nestling stage (a and c). Shown are mean model estimates ± SE.

The number of female forays per hour (*N* = 23 655 observation hours, 26 females) was associated with breeding stage (χ ^2^ = 244.84, *P* < 0.001) and time of day (χ ^2^ = 122.52, *P* < 0.001): females made most forays during the nestling stage, whereas they forayed least often in the early morning (see Figure 2). The duration of female forays (*N* = 10 667 forays, 26 females) was associated with the time of the day (χ ^2^ = 81.87, *P* < 0.001) but not with breeding stage (χ ^2^ = 5.78, *P* = 0.22; see Supplementary Tables S5 and S6). Females made the longest forays during the early morning (see Supplementary Figure S4). The number of different nest-box areas visited by females per day also varied between breeding stages (χ ^2^ = 180.39, *P* < 0.001; see Supplementary Table S9 and Supplementary Figure S5): females visited more different areas during the beginning of the breeding season (prefertile stage and during the fertile days before egg laying) than during later stages.

The number of forays made by males per hour was associated with breeding stage and time of day (breeding stage: χ ^2^ = 318.39, *P* < 0.001, time of day χ ^2^ = 41.02, *P* < 0.001; *N* = 21 135 observation hours, 24 males; see Supplementary Table S7). Male foraying activity was lowest during the prefertile phase of his mate and during the early morning (see Figure 2). The duration of male forays was also associated with time of day but not breeding stage (time of day: χ ^2^ = 24.31, *P* < 0.001; breeding stage: χ ^2^ = 2.83, *P* = 0.59; *N* = 14 419 forays, 24 males; see Supplementary Table S8): forays were longest in the early morning (see Supplementary Figure S4). The duration of male forays in the early morning was not associated with male singing behavior at dawn (PC1: χ ^2^ = 2.01, *P* = 0.16; PC2: χ ^2^ = 2.20, *P* = 0.14; PC3: χ ^2^ = 0.089, *P* = 0.77; *N* = 781 forays, 11 males). Moreover, the number of different nest-box areas visited by males per day was associated with breeding stage (χ ^2^ = 17.0, *P* = 0.002; see Supplementary Table S10), with males visiting the fewer core territories during egg laying and the nestling stage.

### Forays by males and females did not predict the occurrence of extrapair offspring

Extrapair chicks made up 17.7% of the chicks sampled and 40% of broods analyzed contained at least one extrapair chick. The average number of male visits per hour to a nest-box area did not predict the occurrence of extrapair offspring in the visited nest, neither did the average number of forays made per hour by the female or the male of the brood containing extrapair chicks (male visits: *Z* = −0.71, *P* = 0.48; female forays: *Z* = −0.09, *P* = 0.92; male forays: *Z* = 0.41, *P* = 0.69; see Supplementary Tables S11–S13).

## Discussion

Our results integrating movement, signaling, and paternity information reveal that forays by both female and male great tits were associated with the dawn song characteristics of resident males but not with male plumage traits. We show that female forays to other territories were associated with the resident male’s repertoire size and the start and duration of its dawn song (PC1). Male forays, on the other hand, were mainly associated with a male’s song rate and the proportion of time spent singing during the dawn song (PC2). In contrast to our expectations, females stayed away from areas where resident males had a larger repertoire, started their dawn song earlier, and sang for longer at dawn (high PC1 scores), whereas males were attracted to areas where resident males had a high song rate and spent a larger proportion of time singing at dawn (high PC2 score). Additionally, females forayed often throughout the whole tracking period and not primarily when they were fertile. Moreover, neither female nor male foraying was associated with the probability of being cuckolded. These findings, thus, indicate that long-range acoustic (dawn song), but not short-range visual, signals are associated with female and male decisions regarding off-territory movements and, thus, appear to play an important role in connecting individuals within a territorial neighborhood.

Contrary to our predictions, female great tits forayed less often to territories of resident males with a larger repertoire and an earlier and longer dawn song (higher PC1) and tended to make shorter visits to territories of males with a longer song duration (higher PC3). Male song is typically considered to be an honest signal of male quality important in both female attraction and territory defense (Gil and Gahr 2002; Catchpole and Slater 2008). Females engaging in extrapair copulations to gain, for example, indirect genetic benefits for their offspring would, thus, be expected to be attracted to males with more elaborate song traits, such as a larger repertoire (Hasselquist et al. 1996), a more consistent vocal performance (Byers 2007), or an earlier start of the dawn song (Poesel et al. 2006). Female hooded warblers (*Wilsonia citrina*), for example, were more likely to foray off-territory when their social mate sang at a low rate and preferred extrapair mates with a higher song rate (Chiver et al. 2008). However, female preference for more elaborate versions of male song traits are not always evident; many field studies, for example, did not find evidence for female preference for larger repertoire sizes (reviewed in Byers and Kroodsma 2009). Moreover, two recent meta studies found no associations between measures of song complexity and reproductive success (Soma and Garamszegi 2011) and song complexity or output and extrapair paternity both among and within species (Garamszegi 2004). More elaborate song traits are often associated with male territory tenure, dominance, and willingness to escalate an interaction (Krebs et al. 1978; Otter et al. 1997; Vehrencamp 2001), and females may not always prefer more dominant males (Qvarnström and Forsgren 1998; Ophir and Galef 2003; Wong and Candolin 2005). Females in the Atlantic molly (*Poecilia mexicana*), for example, showed a preference for superior fighting abilities but avoided winners after observing male contests, possibly, to avoid harassing behavior of these more dominant males (Bierbach et al. 2013). Thus, it may be possible that females use the information conveyed in dawn singing to avoid aggressive males when foraying. This may be beneficial for females, especially, if they do not foray primarily in search of extrapair mates but for other purposes or if they engage in extrapair mating for reasons other than gaining indirect benefits by mating with higher-quality males. Indeed, previous studies in great tits have found no difference between extrapair mates and social mates in age, body size, survival, and the width of the breast stripe, suggesting that gaining indirect benefits may not be a primary reason for extrapair mating in females (Krokene et al. 1998; Strohbach et al. 1998). Rather, variation in extrapair paternity in this species seems to be connected to consistent behavioral differences between individuals (van Oers et al. 2008; Patrick et al. 2012), which are likely to play an important part in the interactions between females, males, and extrapair males.

Female foraying behavior did not predict the occurrence of extrapair offspring in our study. Moreover, in contrast to other studies (Neudorf et al. 1997; Double and Cockburn 2000; Chiver et al. 2008), we did not find that females were primarily foraying when they were fertile, which we would have expected if females were foraying mostly to encounter potential extrapair mates. Even though females also forayed quite substantially during the fertile days just before egg laying, their foraying activity peaked during chick rearing. Additionally, although females also forayed early in the morning, they forayed mostly later during the day. Foraying early in the morning has previously been suggested to be a foraying strategy to avoid detection (Double and Cockburn 2000) and possibly punishment by the social mate (Westneat and Stewart 2003). Hence, female forays may serve multiple purposes, such as information gathering in a broader sense or foraging in addition to extrapair mating. That females forayed particularly often when having nestlings may suggest that they foray primarily in search of food. However, on forays during the days before egg laying and during egg laying, females could still be prospecting extrapair mates. That females forayed to more different areas during the breeding stages before egg laying might indicate that they are assessing several potential extrapair mates during this early time in the season before narrowing their search down. However, they could also be gathering other types of information during these early forays, for example, on important foraging areas that they could later visit more specifically during chick rearing or just move around more before egg laying and incubation. Future studies could test the foraging function experimentally by supplementing females with food and follow the effect on subsequent foraying effort (Humbird and Neudorf 2008). Moreover, although our data provides almost continuous information on foraying activity of many individuals over the majority of the breeding season, it does not provide information about with whom they interact. More fine-scale spatial information would allow to determine whether foraying birds actually had close-range encounters with the opposite sex member of the resident pair and whether females had more such encounters on forays they made when fertile, which would be expected if they forayed in search of extrapair mates.

In contrast to females, males forayed more often and for a longer time to nest-box areas of resident males with a high song rate and proportion of time spent singing during the dawn song (high PC2). However, males stayed for a shorter time in areas when the resident male sang longer songs during its dawn song (high PC3) and tended to stay for shorter time when males had a large repertoire size and an early and long dawn song (high PC1). More elaborate singing is often associated with male dominance and stronger territory defense and commonly thought to function as a deterrent to conspecific males (Gil and Gahr 2002; Catchpole and Slater 2008). Yet, almost all studies on the keep-out signal function of song are playback studies that simulate an intruder on a territory and measure the immediate response by a territory owner (Catchpole and Slater 2008). Few studies have actually focused on the more cryptic movements of potential intruders and measured whether the broadcast signal keeps them away (Yasukawa 1981; Falls 1988; Naguib et al. 2004; Snijders et al. 2017b), and which song traits might be particularly important in doing so (Krebs et al. 1978; Nowicki et al. 1998). In European starlings (*Sturnus vulgaris*), males were actually attracted to nest-boxes from which male song was broadcast compared with control boxes with no song. Interestingly, these males still preferentially approached boxes where song of lower complexity was broadcast compared with boxes with more complex song, indicating that singing as such could attract males, but differences in song characteristics could still act as a deterrent (Mountjoy and Lemon 1991). In great tits, males with a larger repertoire size may be more successful in keeping intruders out (Krebs et al. 1978) and mean song duration is positively correlated with dominance (Lambrechts and Dhondt 1986). Although we found that males stayed for a shorter time in areas when resident males sang longer songs during their dawn song (high PC3) and tended to stay for a shorter time when males had a high PC1 score (which included repertoire size as measure), males did not visit those areas less often. Males indeed might be attracted to territories of very actively singing males (high PC2 score) to gather information on territory quality or breeding success for future breeding attempts (Doligez et al. 1999; Doligez et al. 2004) as male singing activity can reflect food availability (Ritschard and Brumm 2012). Indeed, male foraying behavior in our study was not associated with the occurrence of extrapair offspring: nests in areas that were visited more by foraying males were not more likely to have extrapair offspring, nor were males that left their territory more often more likely to be cuckolded. Moreover, just like females, males showed a peak in foraying activity during the nestling phase, which could indicate that they foray primarily to forage, although males could copulate with fertile females during the whole period. Males made the longest forays early in the morning, which included the time window of twilight before sunrise when they sing their dawn song. However, the duration of those early morning forays was not associated with any of the male signing measures (PC1, PC2, or PC3). Males that sang very actively and for longer in the morning did, thus, not foray less often during that time, suggesting that males do not trade-off singing with foraying. Together, these results suggest that males foray primarily for reasons other than to seek extrapair copulations.

Our findings that females visited territories of males with a larger repertoire size and an earlier and longer dawn song (high PC1) less often and males visited territories of males with a high song rate and proportion of time spent singing (high PC2) more often contrasts the common view on the function of bird song as a territory defense or female attraction signal. Although many previous studies showed that male bird song in the short term repels males from approaching (Krebs et al. 1978; Nowicki et al. 1998; Snijders et al. 2017a) and attracts females (Snijders et al. 2017a), we here show the opposite by integrating singing with movements at other times of the day and over the whole breeding season. Our results, thus, suggest that male song may influence receiver movements differently at different times of the day and moments during the breeding season beyond a narrow, immediate time window during or right after the actual singing. It is important to note that the long-term spatial response (forays throughout the breeding season) we measured could also reflect within-individual changes in singing behavior throughout the breeding season that we have not captured with measures from one dawn song. However, previous studies in the same population have shown that at least three of the six dawn song measures we used (song rate, start time of dawn song, and repertoire size) were repeatable between some breeding stages (Snijders et al. 2015; Naguib et al. 2019), and other studies have shown that single dawn song recordings can provide important information about the singer (see, e.g., Otter et al. 1997; Poesel et al. 2006).

Taken together, our results show that males and females made frequent forays to other territories; however, forays were neither associated with plumage traits of males nor the occurrence of extrapair offspring. Moreover, although females also forayed often during the fertile days before egg laying and during egg laying, they forayed mostly during chick rearing. Our results do, thus, not provide support for the idea that females use extraterritorial forays to pursue copulations with more elaborately ornamented or singing extrapair mates, although some of the foraying activity during fertile days could still serve this purpose. Our findings that male dawn song was associated with forays of both females and males but in the opposite way than shown by studies on more immediate responses to song is surprising and suggests that, when including long-term responses to signals, there might be additional effects of bird song on receivers. Our findings, including such long-term responses, thus open a new perspective on the role of signaling in spatial and social relations in a population.

## SUPPLEMENTARY MATERIAL

Supplementary data are available at *Behavioral Ecology* online.

araa040_suppl_Supplementary_Information

## Funding

This work was supported by the Dutch Research Council with an ALW open competition grant (grant number: 824.15.012) to M.N.

We would like to thank Geldersch Landschap & Kasteleen for the permission to conduct fieldwork in Westerheide and Mara Ruiz Minano, Maeliss Hoarau, Laura Lute, Tomas Tuvillo, and Hongye Zhang for their help with collecting the data. We are grateful to Piet de Goede, Christa Mateman, Martijn van der Sluijs, Lydia Nieuwe Weme, and the animal care takers at NIOO-KNAW for their valuable assistance throughout the project, John Burt and Hans Meier for the technical support with the Encounternet system, and Liam Bailey for help with the statistical analysis. This study was permitted by the Dutch legal entity Dier Experimenten Comissie no. NIOO-10.05 to M.N. and K.v.O. and no. NIOO-12.02 to K.v.O.

Data accessibility: Analyses reported in this article can be reproduced using the data provided by Bircher et al. (2020).
