# Supplementary material for: Extraterritorial forays by great tits are associated with dawn song in unexpected ways
Source: Behav Ecol. 2020 May 20;31(4):873–83. doi: 10.1093/beheco/araa040 (PMC7390995; doi:10.1093/beheco/araa040)
Supplement: araa040_suppl_Supplementary_Information [file araa040_suppl_supplementary_information.docx]

Supplementary Information Text

Calibration of radio-tracking system

Received signal strength indication (RSSI) values recorded by Encounternet receivers for each detected tag signal decrease with distance (Figure S3 - A). We used these signal strength values to estimate the distance between detected tags and receivers (Mennill et al. 2012). To estimate the relationship between distance and received signal strength we placed four tags at different distances along nine transects within the study site. We choose areas of transects so they encompassed differently vegetated areas in our study site. We measured signal strength values along each transect at 2, 10, 20, 30, 40 and 50m distance to the same receiver. We attached tags to a plastic pole and held the pole in position for six minutes at each distance, placing tags at ground level, 2m and 6m height for two minutes each. To simulate birds in different positions and either moving around or not, we attached tags with their antennas horizontal or perpendicular relative to the receiver’s antenna and turned the pole clockwise with approximately 10 turns during the second minute of measuring at each height. On average tags were detected 95% of the time when within 10 meters and 81% of the time when within 20 meters of a receiver (see Figure S3 – B). We then used a RSSI-distance regression including all transect measures to predict the signal strength value at 15m (obtaining a value of -3.88 RSSI) and used this value to exclude logs determined to be further away than 15m from the receiver at a given nest box. We choose a radius of 15m so we could assign a received signal to just one nest box in most cases (the smallest distance between neighboring boxes in our study area was 30m).

We calibrated all receivers before deployment to account for between receiver variation in detection sensitivity. We attached four tags to a 2m long plastic pole and held the pole at fixed distance of 2m to each of the receivers for two minutes. To account for different antenna angles when tags are on birds, we attached tags on the pole with the antennas of two them positioned parallel and of the other two perpendicular relative to the receiver’s antenna. We then calculated the mean signal strength logged by each receiver (mean*_Receiver_*) and the mean signal strength over all receivers (mean*_RSSI_*). For all receivers with mean*_Receiver_* lower or higher than mean*_RSSI_* ± 1 sd, we adjusted the signal strength value of all logs during analysis by Δ|mean*_RSSI_*-mean*_Receiver_*| to avoid an over or underestimation of the distance between tagged birds and respective receivers.

**Figure S1**

**
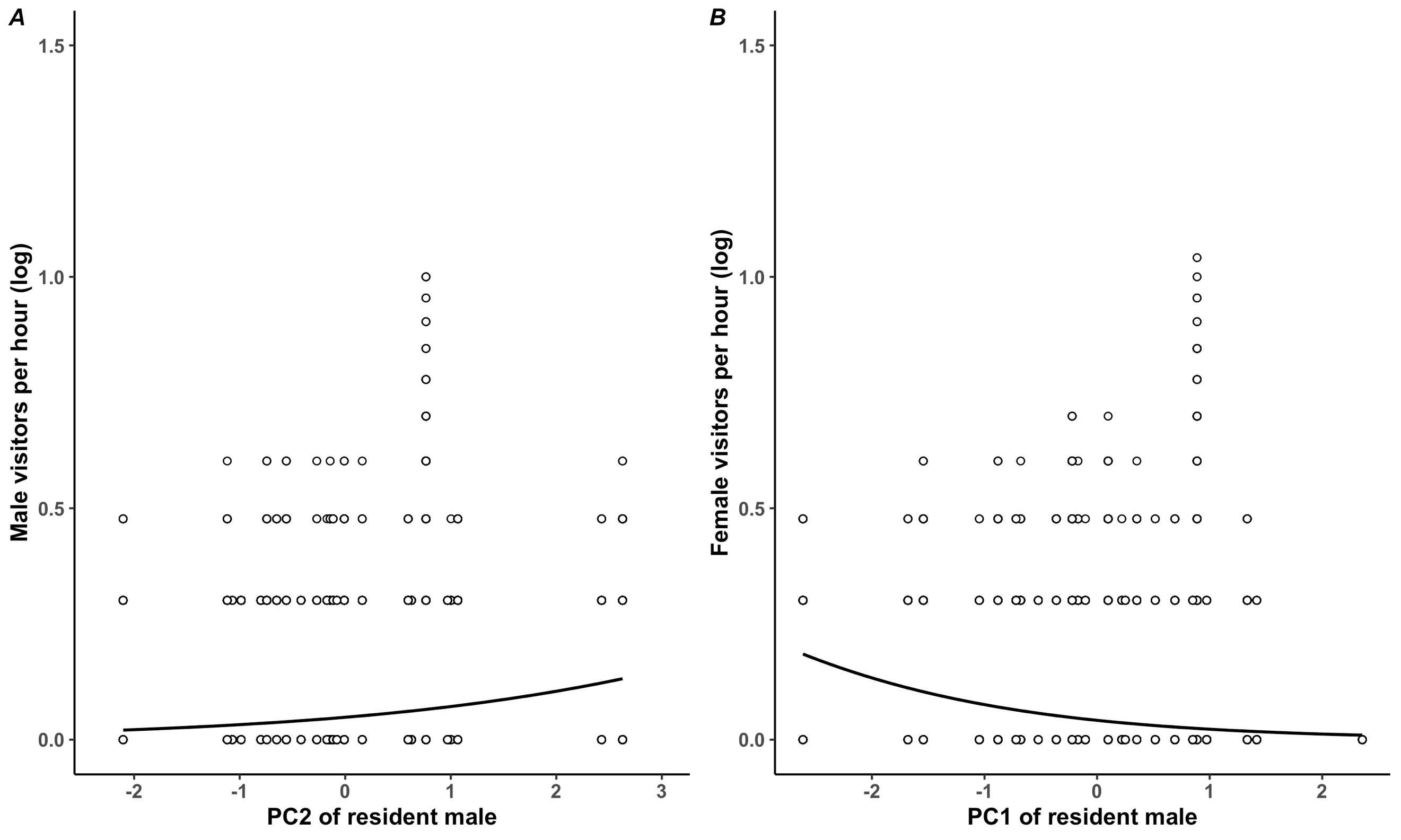
**

**Figure S1 - Associations between song traits (PC1 and PC2) of the resident male and number of male and female visitors to the respective nest box area.** Males with a higher PC2 score were visited by more different males. Males with a higher PC1 score received visits by fewer females. We added 1 to counts of visitors per hour in order to present them on a log scale. Data includes 20,265 observation hours and 24 resident males, observations with the same values are plotted as one point. Lines depict model predictions.

**Figure S2**


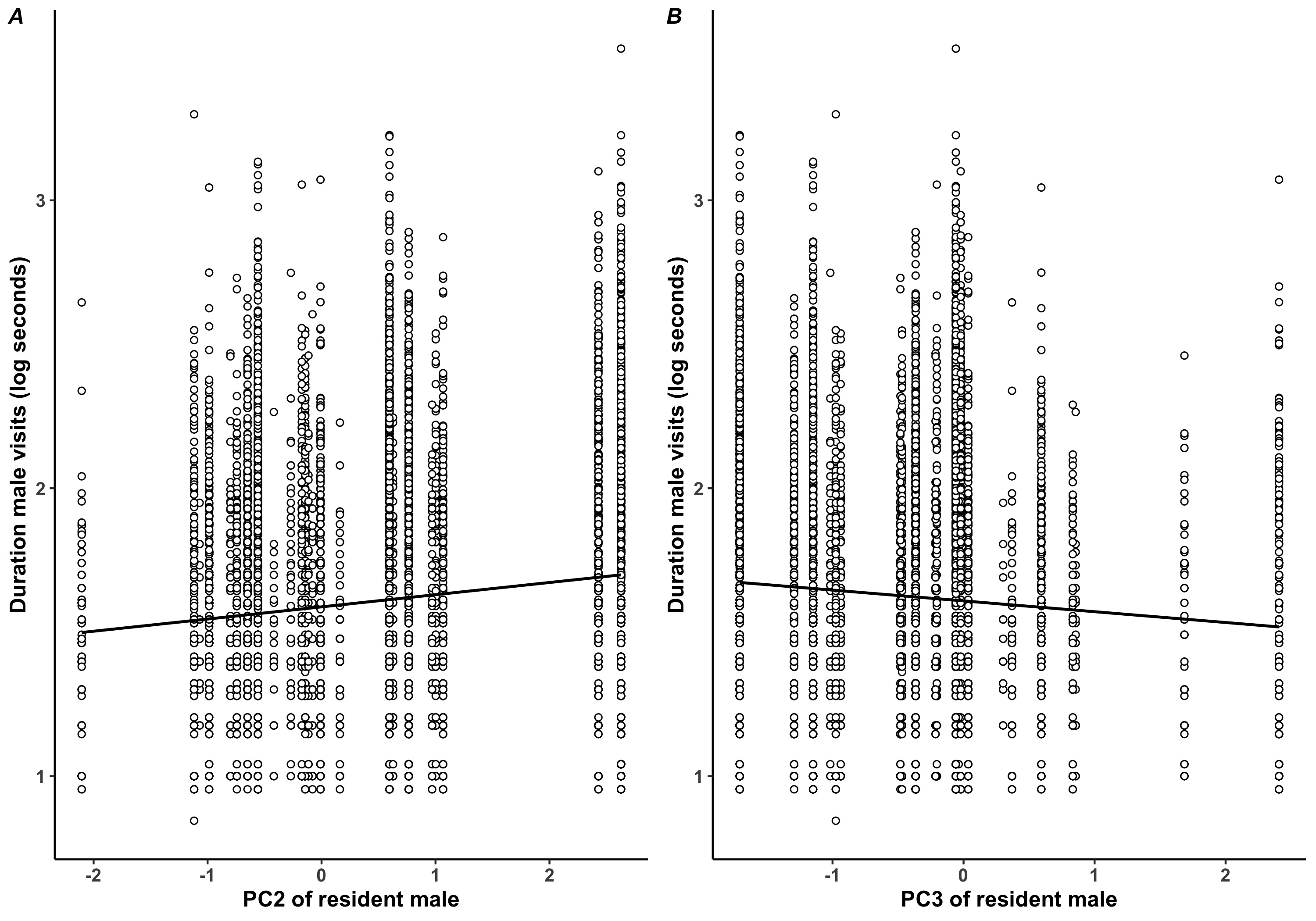


**Figure S2 - Associations between song traits (PC2 and PC3) of the resident male and duration of male visits to the respective nest box area.** Males with a higher PC2 score received longer visits by other males, whereas males with a higher PC3 score received shorter visits. Data include 10,253 observation hours and 24 resident males, observations with the same values are plotted as one point. Lines depict model predictions.

**Figure S3**

**
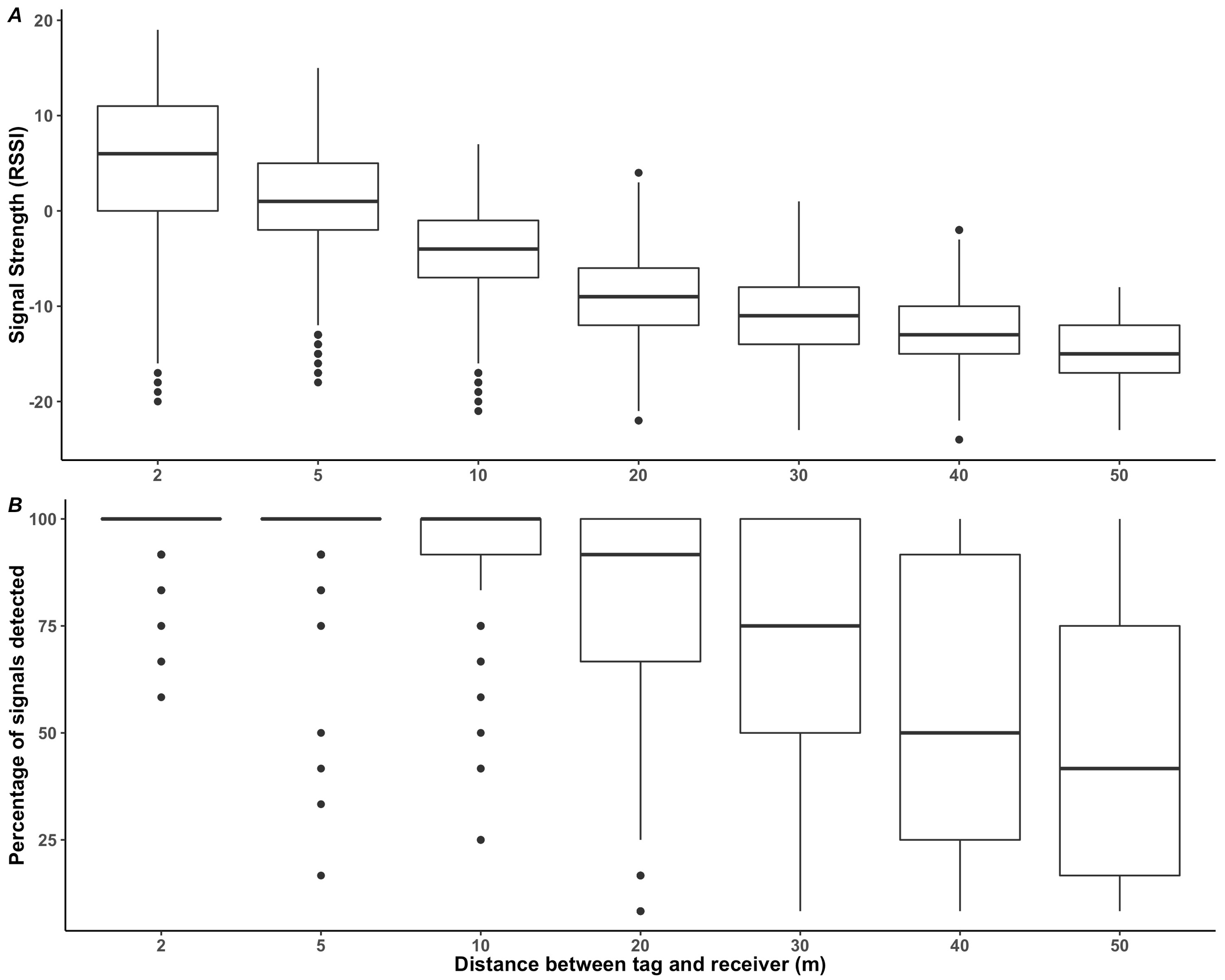
**

**Figure S3 - Signal strength (RSSI), percentage of detected signals and distance between tags and receiver.** Signal strength (A) and percentage of detected signals (B) decreased with the distance between tags and receiver. Data include measures from nine different transects with four tags positioned at three different heights (ground level, 2 and 6m), with two different antenna angles (horizontal and perpendicular) and tags either being moved slightly or held still.


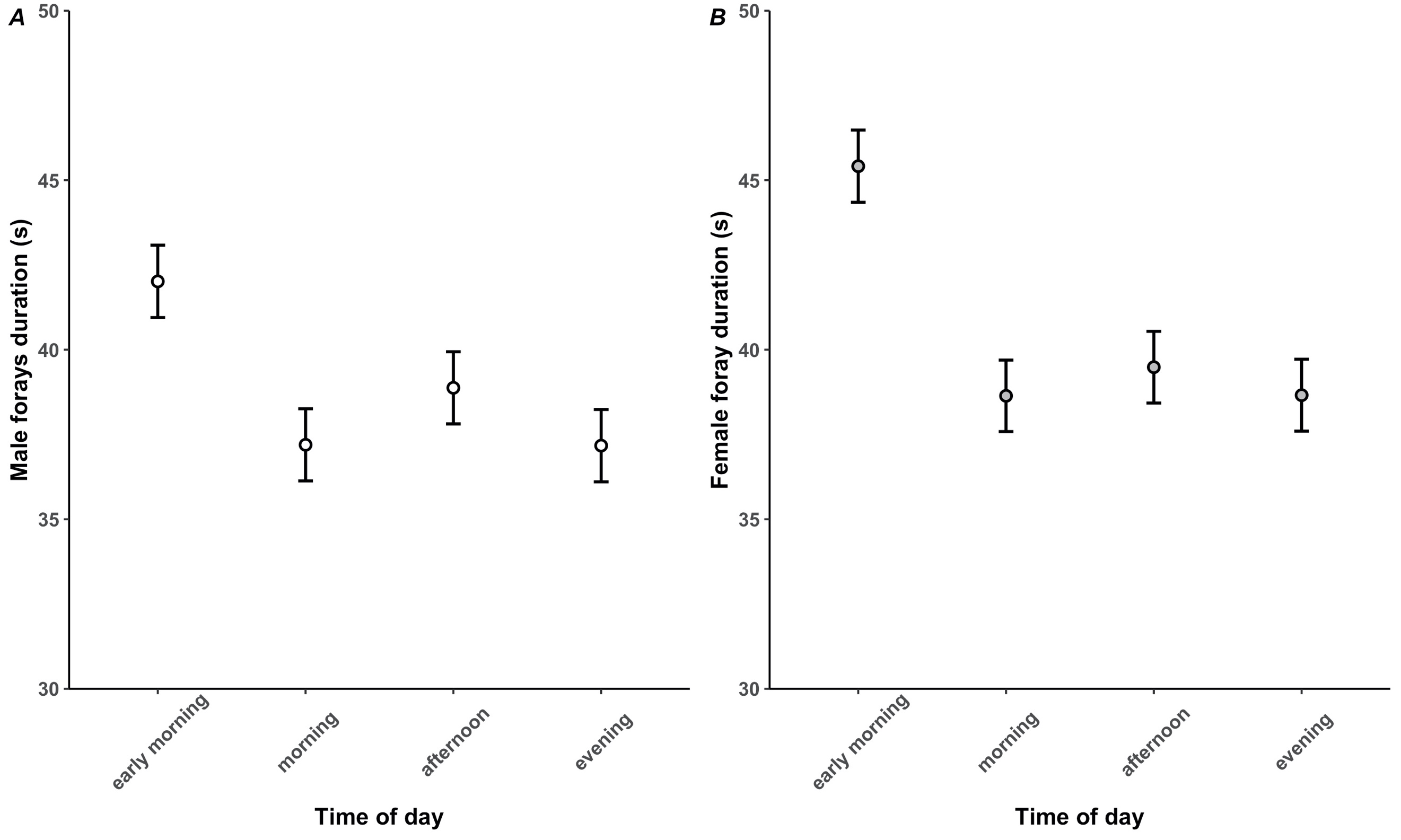


**Figure S4** - **Foray duration of males and females over the day.** Both males (white) and females (grey) made the longest forays early in the morning. Shown are mean model estimates ± se.


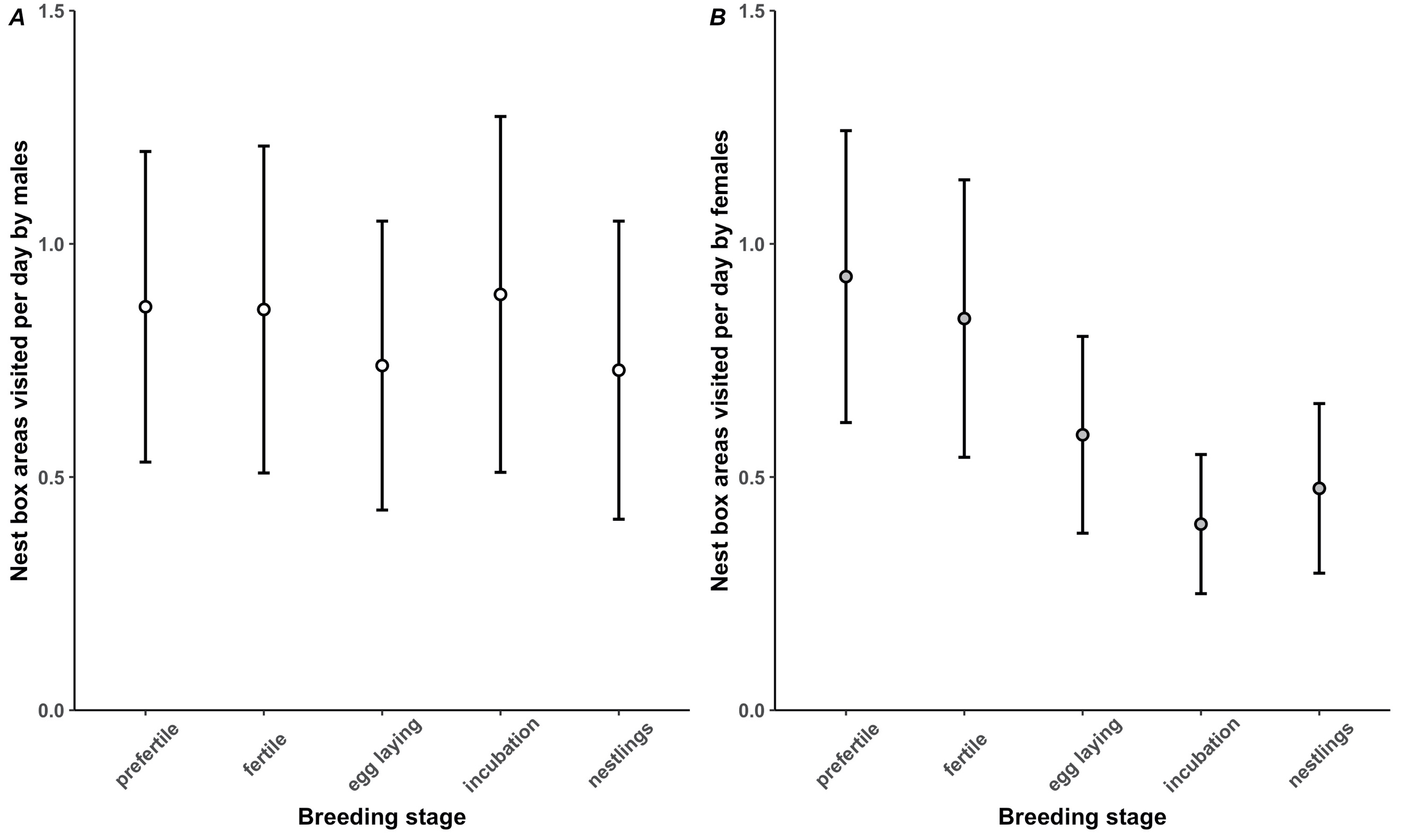


**Figure S5 - Number of nest box areas visited per day by foraying males and females over the breeding season.** Males (white) visited fewer areas during the egg laying and nestling stage. Females (grey) visited more areas early in the season during the prefertile stage and the fertile days before egg laying (fertile). Shown are mean model estimates ± se.

**Table S1: Traits of resident male and number of male visitors per hour.** Table lists all factors included in a poisson generalized linear mixed model with log link function. The dependent variable was the number of male visitors per hour to a resident male’s nest box area (*N* = 20265 observation hours, 24 resident males). Nest box area was included as random factor (var ± sd: 0.84 ± 0.92). Using backward elimination, the estimate and standard error of the last model in which a factor was included are given. The test statistic (χ^2^), degrees of freedom (df) and significance (p value) given were determined using the likelihood-ratio test (LRT). The factors distance (average distance of a resident male’s nest box to other nest boxes) and relative lay date of the resident male’s brood were included in the last model, independent of significance.

**Table S1: Number of male visitors per hour – traits of resident male**

|  | **Estimate** | **se** | **χ^2^** | **df** | **P value** |
| --- | --- | --- | --- | --- | --- |
| Intercept | -2.14 | 0.19 | - | - | - |
| PC2 | 0.42 | 0.18 | 5.04 | 1 | **0.03** |
| Distance | -0.4 | 0.17 | - | - | - |
| Relative lay date | -0.08 | 0.19 | - | - | - |
| *Dropped terms* |  |  |  |  |  |
| Yellowness | -0.03 | 0.20 | 0.02 | 1 | 0.88 |
| PC1 | -0.07 | 0.18 | 0.17 | 1 | 0.68 |
| Stripe | -0.09 | 0.13 | 0.12 | 1 | 0.68 |
| PC3 | 0.41 | 0.17 | 1.33 | 1 | 0.25 |

**Table S2: Traits of resident male and number of female visitors per hour.** Table lists all factors included in a poisson generalized linear mixed model with log link function. The dependent variable was the number of female visitors per hour to a resident male’s nest box area (*N* =20265 observation hours, 24 resident males). Nest box area was included as random factor (var ± sd: 1.10 ± 1.05). Using backward elimination, the estimate and standard error of the last model in which a factor was included are given. The test statistic (χ^2^), degrees of freedom (df) and significance (p value) given were determined using the likelihood-ratio test (LRT). The factors distance (average distance of a resident male’s nest box to other nest boxes) and relative lay date of the resident male’s brood were included in the last model, independent of significance.

**Table S2: Number of female visitors per hour – traits of resident male**

|  | **Estimate** | **se** | **χ^2^** | **df** | **P value** |
| --- | --- | --- | --- | --- | --- |
| Intercept | -1.21 | 2.18 | - | - | - |
| PC1 | -0.64 | 0.22 | 7.72 | 1 | **0.006** |
| Distance *(scaled)* | -2.18 | 4.45 | - | - | - |
| Relative lay date *(scaled)* | -0.01 | 0.06 | - | - | - |
| *Dropped terms* |  |  |  |  |  |
| Yellowness | -0.05 | 0.22 | 0.05 | 1 | 0.82 |
| Stripe | -0.06 | 0.15 | 0.15 | 1 | 0.70 |
| PC2 | 0.39 | 0.22 | 0.72 | 1 | 0.40 |
| PC3 | 0.38 | 0.23 | 2.48 | 1 | 0.12 |

**Table S3: Traits of resident male and duration of male visits.** Table lists all factors included in linear mixed model with the log-transformed duration of male visits to a resident male’s nest box area (*N* = 10253 observation hours, 24 resident males) as dependent variable. Nest box area was included as random factor (var ± sd: 0.002 ± 0.04, residual variance ± sd: 0.16 ±0.39). Using backward elimination, the estimate and standard error of the last model in which a factor was included are given. The test statistic (χ^2^), degrees of freedom (df) and significance (p value) given were determined using the likelihood-ratio test (LRT). The factors distance (average distance of a resident male’s nest box to other nest boxes) and relative lay date of the resident male’s brood were included in the last model, independent of significance.

**Table S3: Duration of male visits – traits of resident male**

|  | **Estimate** | **se** | **χ^2^** | **df** | **P value** |
| --- | --- | --- | --- | --- | --- |
| Intercept | 1.57 | 0.08 | - | - | - |
| PC1 | -0.02 | 0.01 | 3.24 |  | *0.07* |
| PC2 | 0.04 | 0.01 | 12.68 | 1 | **<0.001** |
| PC3 | -0.04 | 0.01 | 7.19 | 1 | **0.007** |
| Distance | 0.09 | 0.17 | - | - | - |
| Relative lay date | -0.01 | 0.003 | - | - | - |
| *Dropped terms* |  |  |  |  |  |
| Yellowness | 0.03 | 0.09 | 0.01 | 1 | 0.92 |
| Stripe | 0.006 | 0.008 | 0.49 | 1 | 0.48 |

**Table S4: Traits of resident male and duration of female visits.** Table lists all factors included in a linear mixed model with the log-transformed duration of female visits to a resident male’s nest box area (*N* = 9430 observation hours, 23 resident males) as dependent variable. Nest box area was included as random factor (var ± sd: 0.004 ± 0.06, residual variance ± sd: 0.15 ±0.38). Using backward elimination, the estimate and standard error of the last model in which a factor was included are given. The test statistic (χ^2^), degrees of freedom (df) and significance (p value) given were determined using the likelihood-ratio test (LRT). The factors distance (average distance of a resident male’s nest box to other nest boxes) and relative lay date of the resident male’s brood were included in the last model, independent of significance.

**Table S4: Duration of female visits – traits of resident male**

|  | **Estimate** | **se** | **χ^2^** | **df** | **P value** |
| --- | --- | --- | --- | --- | --- |
| Intercept | 1.43 | 0.12 | - | - | - |
| PC3 | -0.03 | 0.02 | 3.29 | 1 | *0.07* |
| Distance | 0.37 | 0.22 | - | - | - |
| Relative lay date | -0.0003 | 0.004 | - | - | - |
| *Dropped terms* |  |  |  |  |  |
| Yellowness | .0.05 | 0.12 | 0.18 | 1 | 0.67 |
| Stripe | 0.004 | 0.01 | 0.14 | 1 | 0.71 |
| PC1 | -0.01 | 0.01 | 0.89 | 1 | 0.35 |
| PC2 | 0.01 | 0.01 | 1.16 | 1 | 0.28 |

**Table S5: Number of female forays, breeding stage and time of day.** Table lists all factors included in a zero-inflated poisson generalized linear mixed model with log link function. The dependent variable was the number of forays undertaken per hour by a female (*N* = 23,655 observation hours, 26 females). Female ID was included as random factor (var ± sd: 2.0 ± 1.40). Using backward elimination, the estimate and standard error of the last model in which a factor was included are given. The test statistic (χ^2^), degrees of freedom (df) and significance (p value) given were determined using the likelihood-ratio test (LRT). The factors distance (average distance of a female’s nest box to other nest boxes) and relative lay date of the female’s brood were included in the last model, independent of significance.

**Table S5: Number of female forays per hour – breeding stage and time of day**

|  | **Estimate** | **se** | **χ^2^** | **df** | **P value** |
| --- | --- | --- | --- | --- | --- |
| Intercept | 2.35 | 1.84 | - | - | - |
| Breeding stage |  |  | 244.84 | 4 | **<0.001** |
| *fertile* | 0.21 | 0.05 |  |  |  |
| *nestlings* | 0.46 | 0.05 |  |  |  |
| *incubation* | -0.29 | 0.05 |  |  |  |
| *prefertile* | 0.13 | 0.04 |  |  |  |
| Daytime |  |  | 122.52 | 3 | **<0.001** |
| *early morning* | -0.57 | 0.05 |  |  |  |
| *evening* | -0.06 | 0.03 |  |  |  |
| *morning* | -0.08 | 0.02 |  |  |  |
| Distance | -7.33 | 3.60 | - | - | - |
| Relative lay date | 0.11 | 0.09 | - | - | - |

**Table S6: Duration of female forays, breeding stage and time of day.** Table lists all factors included in a linear mixed model with the log transformed duration of forays undertaken by a female (*N* = 10,667 forays, 26 females) as dependent variable. Female ID was included as random factor (var ± sd: 0.005 ± 0.07, residual variance ± sd: 0.15 ± 0.38). The test statistic (χ^2^), degrees of freedom (df) and significance (p value) given were determined using the likelihood-ratio test (LRT). The factors distance (average distance of a female’s nest box to other nest boxes) and relative lay date of the female’s brood were included in the last model, independent of significance.

**Table S6: Duration of female forays – breeding stage and time of day**

|  | **Estimate** | **se** | **χ^2^** | **df** | **P value** |
| --- | --- | --- | --- | --- | --- |
| Intercept | 1.60 | 0.02 | - | - | - |
| Daytime |  |  | 81.87 | 3 | **<0.001** |
| *early morning* | 0.06 | 0.02 |  |  |  |
| *evening* | -0.01 | 0.01 |  |  |  |
| *morning* | -0.01 | 0.01 |  |  |  |
| Distance *(scaled)* | -0.0004 | 0.01 | - | - | - |
| Relative lay date *(scaled)* | -0.01 | 0.02 | - | - | - |
| *Dropped terms* |  |  |  |  |  |
| Breeding stage |  |  | 5.78 | 4 | 0.22 |

**Table S7: Number of male forays, breeding stage and time of day.** Table lists all factors included in a zero-inflated poisson generalized linear mixed model with log link function. The dependent variable was the number of forays undertaken per hour by a male (*N* =21,135 observation hours, 24 males). Male ID was included as random factor (var ± sd: 2.8 ± 1.67). Using backward elimination, the estimate and standard error of the last model in which a factor was included are given. The test statistic (χ^2^), degrees of freedom (df) and significance (p value) given were determined using the likelihood-ratio test (LRT). The factors distance (average distance of a male’s nest box to other nest boxes) and relative lay date of the male’s brood were included in the last model, independent of significance.

**Table S7: Number of male forays per hour – breeding stage and time of day**

|  | **Estimate** | **se** | **χ^2^** | **df** | **P value** |
| --- | --- | --- | --- | --- | --- |
| Intercept | 7.12 | 2.65 | - | - | - |
| Breeding stage |  |  | 318.39 | 4 | **<0.001** |
| *fertile* | -0.02 | 0.04 |  |  |  |
| *chick rearing* | 0.24 | 0.05 |  |  |  |
| *incubation* | 0.20 | 0.03 |  |  |  |
| *prefertile* | -0.25 | 0.03 |  |  |  |
| Daytime |  |  | 41.02 | 3 | **<0.001** |
| *early morning* | -0.22 | 0.04 |  |  |  |
| *evening* | -0.01 | 0.03 |  |  |  |
| *morning* | -0.02 | 0.02 |  |  |  |
| Distance | -17.0 | 6.0 | - | - | - |
| Relative lay date | 0.01 | 0.08 | - | - | - |

**Table S8: Duration of male forays, breeding stage and time of day.** Table lists all factors included in a linear mixed model with log transformed duration of forays undertaken by a male (*N* = 14,419 forays, 24 males) as dependent variable. Male ID was included as random factor (var ± sd: 0.005 ± 0.07, residual variance ± sd: 0.15 ± 0.38). The test statistic (χ^2^), degrees of freedom (df) and significance (p value) given were determined using the likelihood-ratio test (LRT). The factors distance (average distance of a male’s nest box to other nest boxes) and relative lay date of the male’s brood were included in the last model, independent of significance.

**Table S8: Duration of male forays – breeding stage and time of day**

|  | **Estimate** | **se** | **χ^2^** | **df** | **P value** |
| --- | --- | --- | --- | --- | --- |
| Intercept | 1.59 | 0.02 | - | - | - |
| Daytime |  |  | 24.31 | 3 | **<0.001** |
| *early morning* | 0.04 | 0.01 |  |  |  |
| *evening* | -0.02 | 0.01 |  |  |  |
| *morning* | -0.02 | 0.01 |  |  |  |
| Distance *(scaled)* | -0.02 | 0.02 | - | - | - |
| Relative lay date *(scaled)* | -0.01 | 0.02 | - | - | - |
| *Dropped terms* |  |  |  |  |  |
| Breeding stage |  |  | 2.83 | 4 | 0.59 |

**Table S9: Number of nest box areas visited per day by females and breeding stage.** Table lists all factors included in a poisson generalized linear mixed model with log link function. The dependent variable was the number of nest box areas visited per day by a female (*N* = 1584 observation days, 26 females). Female ID was included as random factor (var ± sd: 1.0 ± 1.0). Using backward elimination, the estimate and standard error of the last model in which a factor was included are given. The test statistic (χ^2^), degrees of freedom (df) and significance (p value) given were determined using the likelihood-ratio test (LRT). The factors distance (average distance of a female’s nest box to other nest boxes) and relative lay date of the female’s brood were included in the last model, independent of significance.

**Table S9: Number of nest box areas visited by females per day – breeding stage**

|  | **Estimate** | **se** | **χ^2^** | **df** | **P value** |
| --- | --- | --- | --- | --- | --- |
| Intercept | -0.68 | 0.22 | - | - | - |
| Breeding stage |  |  | 180.39 | 4 | **<0.001** |
| *fertile* | 0.38 | 0.11 |  |  |  |
| *nestlings* | -0.26 | 0.14 |  |  |  |
| *incubation* | -0.47 | 0.12 |  |  |  |
| *prefertile* | 0.52 | 0.09 |  |  |  |
| Distance *(scaled)* | -0.38 | 0.20 | - | - | - |
| Relative lay date *(scaled)* | 0.33 | 0.20 | - | - | - |

**Table S10: Number of nest box areas visited per day by males and breeding stage.** Table lists all factors included in a poisson generalized linear mixed model with log link function. The dependent variable was the number of nest box areas visited per day by a male (*N* = 1417 observation days, 24 males). Male ID was included as random factor (var ± sd: 1.26 ± 1.12). Using backward elimination, the estimate and standard error of the last model in which a factor was included are given. The test statistic (χ^2^), degrees of freedom (df) and significance (p value) given were determined using the likelihood-ratio test (LRT). The factors distance (average distance of a female’s nest box to other nest boxes) and relative lay date of the female’s brood were included in the last model, independent of significance.

**Table S10: Number of nest box areas visited by males per day – breeding stage**

|  | **Estimate** | **se** | **χ^2^** | **df** | **P value** |
| --- | --- | --- | --- | --- | --- |
| Intercept | -0.50 | 0.25 | - | - | - |
| Breeding stage |  |  | 17.0 | 4 | **0.002** |
| *fertile* | 0.19 | 0.10 |  |  |  |
| *nestlings* | -0.12 | 0.13 |  |  |  |
| *incubation* | 0.21 | 0.10 |  |  |  |
| *prefertile* | 0.21 | 0.08 |  |  |  |
| Distance *(scaled)* | -0.64 | 0.25 | - | - | - |
| Relative lay date *(scaled)* | 0.10 | 0.25 | - | - | - |

**Table S11: Occurrence of extra-pair offspring and average number of male visits per hour.** The table consists of all factors included in a binomial generalized linear model with occurrence of extra-pair offspring (0/1) as the dependent variable (*N* = 37 broods). Given is the estimate, test statistic (Z value) and significance (p value).

**Table S11: Occurrence of extra-pair offspring – average number of male visits per hour**

|  | **Estimate** | **se** | **Z value** | **P value** |
| --- | --- | --- | --- | --- |
| Intercept | 1.72 | 2.24 | - | - |
| Average male visits | -0.23 | 0.36 | -0.71 | 0.48 |
| Distance | -3.34 | 4.46 | -0.75 | 0.45 |
| Relative lay date | -0.003 | 0.07 | -0.046 | 1.0 |

**Table S12: Occurrence of extra-pair offspring and average number of forays by the resident male per hour.** The table consists of all factors included in a binomial generalized linear model with occurrence of extra-pair offspring (0/1) as the dependent variable (*N* = 22 broods). Given is the estimate, test statistic (Z value) and significance (p value).

**Table S12:** **Occurrence of extra-pair offspring – average number of male forays per hour**

|  | **Estimate** | **se** | **Z value** | **P value** |
| --- | --- | --- | --- | --- |
| Intercept | -3.86 | 3.9 | - | - |
| Average male forays | 0.14 | 0.35 | 0.41 | 0.69 |
| Distance | 6.92 | 8.98 | 0.77 | 0.44 |
| Relative lay date | -0.03 | 0.11 | -0.30 | 0.77 |

**Table S13: Occurrence of extra-pair offspring and average number of forays by the resident female per hour.** The table consists of all factors included in a binomial generalized linear model with occurrence of extra-pair offspring (0/1) as the dependent variable (*N* = 23 broods). Given is the estimate, test statistic (Z value) and significance (p value).

**Table S13:** **Occurrence of extra-pair offspring – average number of female forays per hour**

|  | **Estimate** | **se** | **Z value** | **P value** |
| --- | --- | --- | --- | --- |
| Intercept | 0.48 | 2.83 | - | - |
| Average female forays | -0.06 | 0.61 | -0.09 | 0.92 |
| Distance | -1.0 | 5.44 | -0.19 | 0.85 |
| Relative lay date | -0.10 | 0.13 | -0.75 | 0.45 |
